# Tuning Hierarchical Ferric Nanostructures-Decorated Diatomite for Supercapacitors

**DOI:** 10.1186/s11671-018-2822-9

**Published:** 2018-12-18

**Authors:** Ming Hao Wu, Kai Lin Li, Xin Yu Zhang, Ping Gan, Jia Lin Ge, Dan Ning Tian, De Bin Jiang, Xiao Ying Liu, Yu Xin Zhang

**Affiliations:** 1 State Key Laboratory of Mechanical Transmissions, College of Materials Science and Engineering, Chongqing University, Chongqing, 400044 People’s Republic of China; 2 School of Microelectronics and Communication Engineering, Chongqing University, Chongqing, 400044 People’s Republic of China; 3 Xicheng Experimental School of the Second High School Attached to Beijing Normal University, Beijing, 100011 People’s Republic of China; 4 High School Affiliated to Southwest University, Chongqing, 400700 People’s Republic of China; 5 Engineering Research Center for Waste Oil Recovery Technology and Equipment of Ministry of Education, College of Environment and Resources, Chongqing Technology and Business University, Chongqing, 400067 People’s Republic of China; 6 School of Foreign Languages and Cultures, Chongqing University, Chongqing, 400044 People’s Republic of China; 7 College of Arts, Chongqing University, Chongqing, 400044 People’s Republic of China

**Keywords:** Porous materials, Nanocomposites, Hierarchical structure, Supercapacitors

## Abstract

FeOOH nanosheets on porous diatomite have been successfully prepared by a facile two-step hydrothermal approach for supercapacitors, and then α-Fe_2_O_3_ and γ-Fe_2_O_3_ nanostructures are obtained via calcination under different atmospheres and temperatures. The morphologies and structures of all the samples are investigated in detail to make the hierarchical architecture clear. Besides, systemic tests are carried out in 1 M Na_2_SO_4_ electrolyte to characterize the electrochemical properties of these materials. Among the iron-related composite electrodes, diatomite@FeOOH owns the highest specific capacitance (157.9 F g^−1^ at a current density of 0.5 A g^−1^) and best cycling performance (98.95% retention after 1000 cycles), which is considered to be a potential material for high-performance supercapacitors. Furthermore, the synthesizing strategy can be extended to the preparation of other metallic oxide-derived functional materials towards energy storage and conversion.

**Electronic supplementary material:**

The online version of this article (10.1186/s11671-018-2822-9) contains supplementary material, which is available to authorized users.

## Background

Up to now, major challenges for supercapacitor technologies include low-energy density and high production cost. Some research efforts have been devoted to improving its disadvantages [1]. Some transition metal oxides or hydroxides, such as MnO_2_ [2–4], FeOOH [5], NiO [6], and CuO [7], are regarded as potential candidates for active electrode materials. Among these transition metal oxides, ferric oxides/hydroxides have drawn considerable attention due to their nature abundance, variable oxidation states and environmental friendliness [8–10]. Besides, ferric oxides/hydroxides have been considered as especially desirable electrode materials for supercapacitors because its structure (like tunnel-type FeOOH) can accelerate ion transport. However, ferric oxides/hydroxides still own two major obstacles (small surface area and low electro-conductivity). Nanostructures can change the obstacles and provide enormous advantages in energy storage system, which are considered to be high charge-discharge rates by accelerating high specific surface areas, fast redox reactions, and short diffusion paths for electrons and ion [11]. Nevertheless, researches indicated that ferric oxide nanostructures had a tendency to get aggregate and transform into large particles causing a severe loss of specific surface area, which has a seriously terrible effect on electrochemical properties [12]. Therefore, the primary issue currently is to find a simple and feasible way to effectively disperse the nanostructures of ferric oxides, such as fabricating ferric metal oxides on the surface of porous templates.

As an important natural porous nanomaterial, diatomite is an attractive porous template on account of its high porosity, low-volume density, stable chemical property, and large specific area [13–15]. Diatomite template can increase the low surface area and avoid aggregation of nanostructures. Until now, despite that the characteristics of their structures are obvious and promising, ferric oxides/hydroxides-based diatomite composites to form a hierarchically porous structure have rarely yet to be studied in supercapacitors.

In this paper, we report successful fabrication of ferric oxides/hydroxides on diatomite as supercapacitor electrode materials by an effective two-step hydrothermal approach. We aimed to use diatomite as a template to distribute evenly ferric oxide/hydroxide nanostructures on the diatomite surface, which solves the problem on how to effectively disperse its nanostructures and greatly improve the surface area and electrochemical properties of ferric oxides/hydroxides, as shown in Additional file 1: SI-5. Remarkably, with diatomite and ferric oxides/hydroxides’ synergistic effect, the final composite, diatomite@FeOOH, indicated promising electrochemical properties in supercapacitors.

## Experimental Section

### Materials Synthesis

**Fig. 1:**
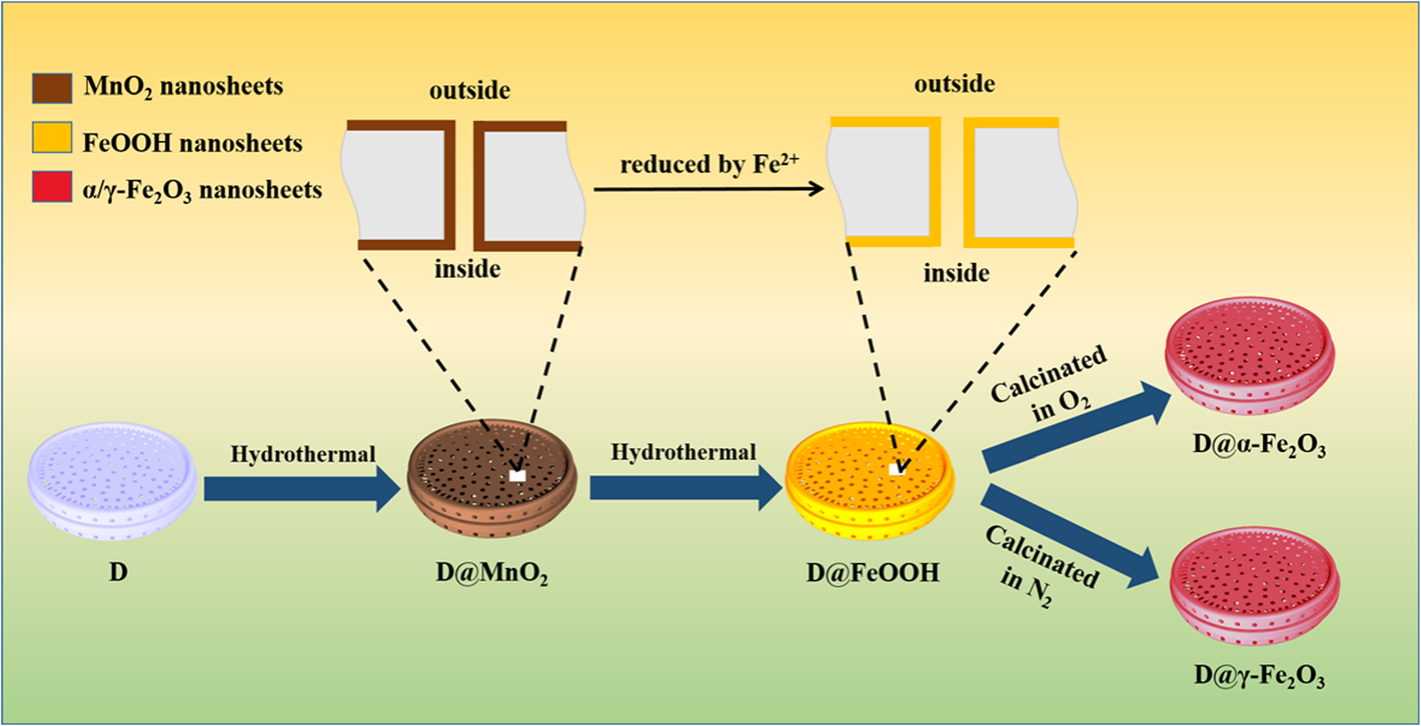
Preparative route of ferric oxides/hydroxides-based diatomites

All the chemical reagents were of analytical purity and used without any further purification. Synthetic processes are as follows (Fig. 1; more details in Additional file 1: SI-1).

Initially, the natural diatomite was purified via a simple oil bath method by the following procedures. Then, the MnO_2_-decorated diatomite were prepared by a hydrothermal method. Typically, the KMnO_4_ solution (30 mL, 0.05 M) was mixed with the purified diatomite (30 mg). Afterwards, the mixture was transferred into a Teflon-lined autoclave (50 mL) which was heat-treated at 160 °C for 24 h. The as-prepared diatomite was centrifuged, washed by distilled water and then dried at 60 °C. After that, MnO_2_-decorated diatomite was obtained.

In addition, a solution of FeSO_4_·7H_2_O (0.01 M, 30 mL) was applied towards totally transformation of the pretreated diatomite@MnO_2_ (30 mg) at 120 °C for 2 h. In the end, the different crystal forms (α-Fe_2_O_3_ and γ-Fe_2_O_3_) of ferric oxide-decorated diatomite were prepared by calcining at 350 °C for 2 h under O_2_ atmosphere and 500 °C for 2 h under N_2_ atmosphere, respectively.

### Characterization

Focused ion beam scanning electron microscopy (Zeiss Auriga FIB/SEM) was employed for observing the morphologies. And the phase analysis and structure were established by powder X-ray diffraction (XRD; D/max 2500, Cu Kα).

### Electrochemical Measurements

Electrochemical study on the materials in a three-electrode system: All the electrochemical properties of the as-obtained diatomite composites were characterized though a conventional three-electrode equipment filled with 1 M Na_2_SO_4_ electrolyte. Before the measurements, the working electrodes were formed with mixing active material (diatomite@MnO_2_, diatomite@FeOOH, diatomite@α-Fe_2_O_3_, and diatomite@γ-Fe_2_O_3_), acetylene black, and polyvinylidene fluoride (PVDF) at a weight ratio of 7:2:1 in *N*-methyl-2-pyrrolidone (NMP). The slurry was coated on pieces of foamed nickel foam (1 × 1 cm^2^), which was heated to evaporate the dissolvent (120 °C for 12 h). About 2 mg electrode material was loaded on the nickel foam. The electrochemical performances and capacitance values of the composites electrodes were characterized with cyclic voltammetry (CV), galvanostatic charging/discharging (CC) methods, and electrochemical impedance spectroscopy (EIS).

The specific capacitance (*C*_*m*_) is calculated by the following equation:

$$ {C}_m=\frac{I\Delta t}{m\Delta V} $$

where *I* is the discharging current, *△t* is the discharging time, *△V* is the potential window during discharging, and *m* is the weight of active materials.

## Results and Discussion

**Fig. 2:**
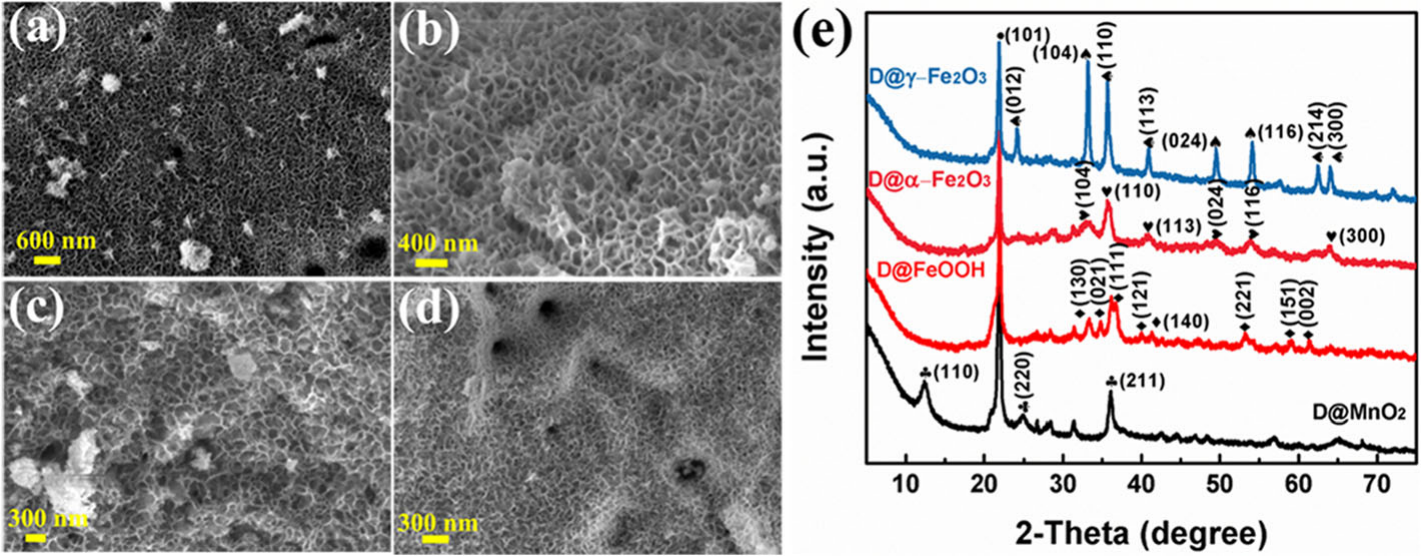
SEM images of diatomite@MnO_2_ nanocomposite (**a**), diatomite@FeOOH nanocomposite (**b**), diatomite@α-Fe_2_O_3_ nanocomposite (**c**), diatomite@γ-Fe_2_O_3_ nanocomposite (**d**); XRD pattern of the four samples (**e**)

Figure 2 presents SEM images of MnO_2_, FeOOH, and α-Fe_2_O_3_/γ-Fe_2_O_3_ nanoarrays on the diatomite. Figure 2a show the uniform and discrete MnO_2_ nanosheets (diatomite@MnO_2_) grown on the diatomite via a facile hydrothermal method. By virtue of acid treatment and calcination, MnO_2_ can combine with diatomite firmly by the interaction force, which facilitates reactions between MnO_2_ and Fe^2+^. Meanwhile, plenty pores of diatomite increase the diffusion of ions. Figure 2b exhibits diatomite@FeOOH have similar morphology compared with MnO_2_ arrays. Indeed, MnO_2_ is reduced by Fe^2+^ ions, and Fe^2+^ ions in solution take the place of Mn. In addition, the pretreatment for stabilization of the crystal MnO_2_ and the assistance of ethylene glycol probably generate the similar nanosheets morphology. The size of α-Fe_2_O_3_ nanosheets (Fig. 2c) is bigger, and the distance between the sheets is larger under the same high-magnification condition, compared with that of γ-Fe_2_O_3_ (Fig. 2d). The morphology of the samples in low magnification can be seen in Additional file 1: SI-2(a–d). Additionally, Additional file 1: SI-2(e, f) exhibit the corresponding EDS mappings of diatomite@MnO_2_ and diatomite@Fe_2_O_3_ and further prove the existence of convictive elements (Mn, Fe, and O), confirming the formation of MnO_2_ and Fe_2_O_3_ nanosheets. Besides, Additional file 1: SI-2(f) shows that there is no Mn element existing in the FeOOH loaded on diatomite, indicating that the MnO_2_ nanosheets were totally transferred into iron hydroxides.

XRD patterns of the as-obtained samples are exhibited in Fig. 2e to confirm the phase composition and structure of the products. It is noted that the strongest peaks of the four samples marked with dot symbol in all the curves are the characteristic peaks of diatomite substrate. The diatomite@MnO_2_ sample showed diffraction peaks at 2*θ* = 12.784°, 25.711°, and 37.522°, corresponding to the (110), (220), and (211) crystal planes (JCPDS card no. 44-0141). As for FeOOH nanosheet arrays, three diffraction peaks of the MnO_2_ disappear in the red curve, while a few well-defined diffraction peaks are well consistent with the standard XRD pattern of FeOOH (JCPDS card no. 29-0713), matching with the (130), (021), (111), (121), (140), (221), (151), and (002) plane. The XRD results of the ferric oxide/hydroxide samples show that the MnO_2_ peaks disappeared and reveal that there are no MnO_2_ nanosheets existing. Therefore, both EDS mapping and XRD results reveal that MnO_2_ is completely replaced by Fe^2+^ ions in this work. Moreover, the diffraction peaks of α-Fe_2_O_3_ are weaker than that of γ-Fe_2_O_3_ about 24.138° and 62.449°, assigned to the (012) and (214) planes of hematite-type ferric oxide crystal (both are JCPDS card no. 33-0664). It confirms again that the replacement between MnO_2_ and Fe^2+^ ions successfully occur at the interfaces of the diatomite and solution.

To investigate the electrochemical properties of the four samples, a three-electrode system was carried out in 1 M Na_2_SO_4_ aqueous electrolyte. The differences in morphologies and structures of these four samples can lead to diverse electrochemical performances. Diatomite served as a substrate contributes to efficient transport of ions owing to its porous structures.

As shown in Additional file 1: SI-3(a, b), the CV and CC curves of diatomite@MnO_2_ electrode are quasi-rectangular and nearly symmetrical triangular, respectively. There are no distinct redox peaks, which deviates from the ideal rectangle manifesting faradic pseudocapacitive nature of the electrode. As revealed in Additional file 1: SI-3(c, d), diatomite@FeOOH has the better capacitive properties than other two samples (diatomite@α/γ-Fe_2_O_3_). The specific capacitance of diatomite@FeOOH electrodes is about 157.9 F g^−1^ at a current density of 0.5 A g^−1^, demonstrating that the highly porous structure can transfer more ions into its surface and can promote more redox faradic reactions. Agreeing with the SEM results, the distances of α-Fe_2_O_3_ nanosheets are so large that the surface of active material make less use of the cations, while γ-Fe_2_O_3_ can provide the smallest specific area for ions among the three ferric oxide samples. Therefore, the distance of the nanosheets of the samples is very important. Besides, as showed in Table 1, the diatomite@FeOOH electrode in this work has a higher specific capacitance among these ferric oxide/hydroxide-based electrodes compared with previous work.

**Table 1:** Comparison of electrochemical performance for the ferric oxide/hydroxide based electrodes

Ferric oxide/hydroxide-based electrode	Electrolyte	Potential range (vs. SCE)	Specific capacitance (F g^−1^)	Ref (year)
FeOOH nanoparticles	1 M Li_2_SO_4_	− 0.85 to − 0.1 V	148 at 0.5 A g^−1^	[16] (2014)
FeOOH nanorods	1 M Li_2_SO_4_	− 0.85 to − 0.1 V	116 at 0.5 A g^−1^	[17] (2008)
FeOOH@MnO_2_ core-shell	1 M LiOH	− 0.15 to 0.6 V	178.6 at 0.1 A g^− 1^	[18] (2017)
Porous flower-like Fe_2_O_3_	0.5 M Na_2_SO_3_	− 0.8 to 0 V	127 at 1 A g^−1^	[19] (2013)
Fe_2_O_3_ sheets	1 M Li_2_SO_4_	− 0.8 to − 0.2 V	147 at 0.36 A g^−1^	[20] (2014)
Fe_3_O_4_ nanosheets/carbon nanofibers	1 M Na_2_SO_3_	− 0.9 to 0.1 V	127 at 10 mV s^−1^	[21] (2011)
Diatomite@FeOOH	1 M Na_2_SO_4_	− 1 to 0 V	157.9 at 0.5 A g^−1^	This work

**Fig. 3:**
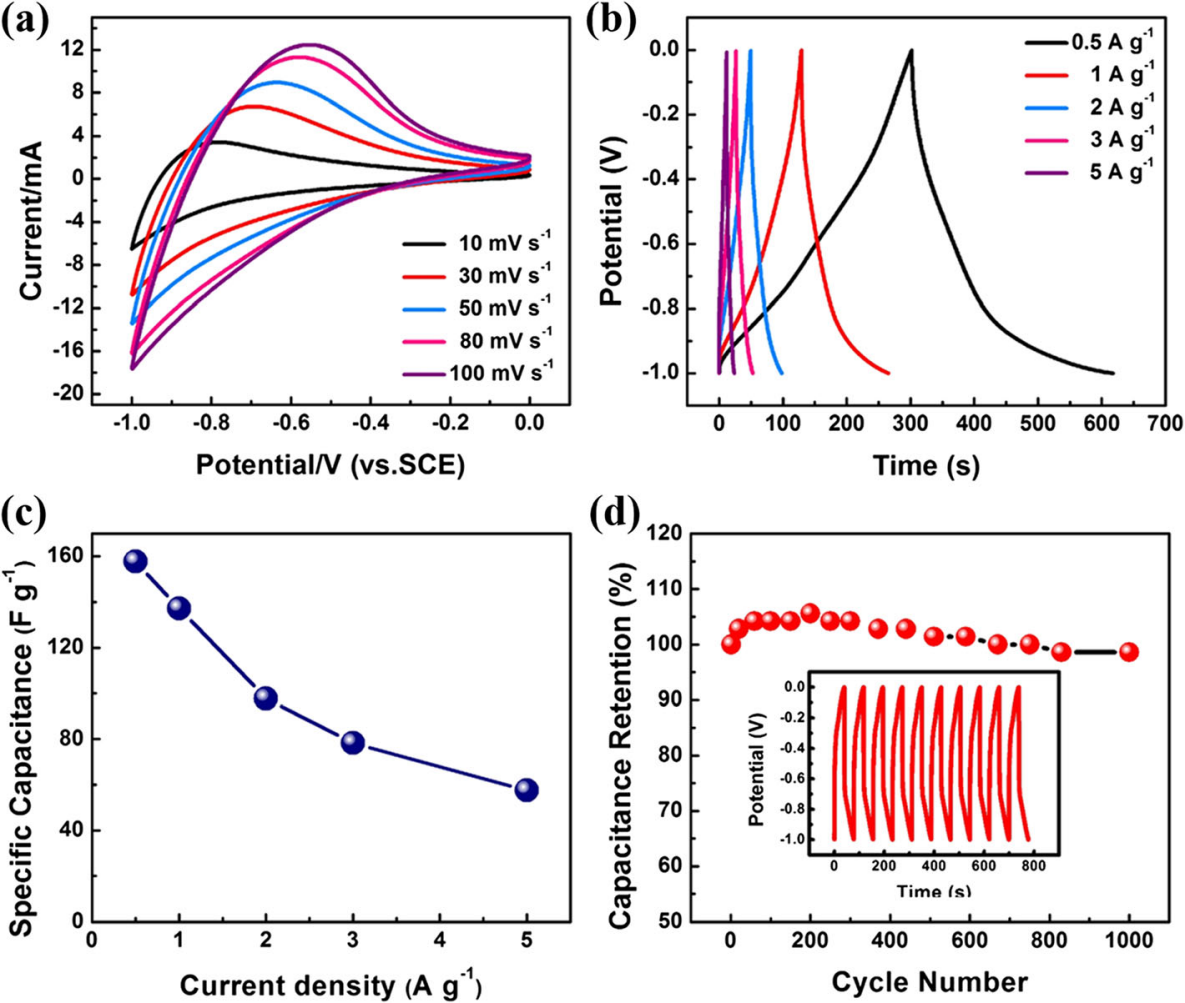
**a** CV curves of Diatomite@FeOOH measured at different scan rates. **b** CC curves of Diatomite@FeOOH measured at different current densities. **c** Specific capacitance measured at different current densities. **d** Cycling performance of the electrode at a current density of 1 A g^−1^ (the inset shows charge-discharge curves of the last 10 cycles)

Such being the case, systematic tests are carried out to better investigate the electrochemical properties of diatomite@FeOOH electrode. Figure 3a shows typical CV curves of FeOOH sample in potential range from − 1 to 0 V at different scan rates. Galvanostatic charge-discharge curves of diatomite@FeOOH electrode at different current densities are presented in Fig. 3b. The shape of CV and CC curves of diatomite@FeOOH electrode demonstrates the pseudocapacitance characteristics of diatomite@FeOOH. Figure 3c further illustrates the relationship between specific and current density. The cycling ability of diatomite@FeOOH electrode was subjected to a long-cycle test for consecutive 1000 cycles (Fig. 3d), and capacity retention after 1000 cycles is about 98.95%. The CC curves of the last 10 cycles suggest no major structure variation during the charge-discharge processes. Additionally, the Nyquist plots for the FeOOH sample electrode (Additional file 1: SI-4) contain a semicircle in the high-frequency boundary and a straight line in the low-frequency range. The internal resistance (*R*_s_) of the electrode is about 3.0 Ω and 3.5 before and after 1000 cycles without much variation, while the charge-transfer resistance (*R*_ct_) is about 1.2 and 4.0 Ω before and after 1000 cycles. These findings could be responsible for the good electrochemical properties of the diatomite@FeOOH electrode.

## Conclusions

In summary, we prepare ferric oxides-decorated diatomite combined with a subsequent replacement process by a facile and effective hydrothermal approach. These ferric oxides/hydroxides own finely controlled morphologies and nanosheet structures. Diatomite@FeOOH material exhibits promising electrochemical properties, which is superior to the other ferric oxide materials. The specific capacitance of diatomite@FeOOH is 157.9 F g^−1^ at a current density of 0.5 A g^−1^, and its cycle performance is good (98.95% retention after 1000 cycles). Actually, the hierarchical and porous diatomite@FeOOH could be a promising active material for supercapacitors. Furthermore, such synthesizing strategy can be extended to the preparation of other metallic oxide-derived functional materials towards energy storage and conversion.

## Additional File

Additional file 1: Experimental section. **Figure S1.** The SEM images of the samples in low magnification. **Figure S2.** (a) CV and (b) CC curves of the diatomite@MnO_2_ in 1 M Na_2_SO_4_; (c) CV and (d) CC curves of three kinds of ferric oxides-decorated diatomite. **Figure S3.** The EIS curve of Diatomite@FeOOH. **Figure S4.** The CV (a) curves at 100 mV s^−1^ and CC (b) curves at 1 A g^−1^ of diatomite, FeOOH and D@FeOOH. (DOC 22468 kb)

## Abbreviations

CC: Galvanostatic charging/discharging
CV: Cyclic voltammetry
EIS: Electrochemical impedance spectroscopy
FIB/SEM: Focused ion beam scanning electron microscopy
NMP: *N*-methyl-2-pyrrolidone
PVDF: Polyvinylidene fluoride
XRD: Powder X-ray diffraction
