# Supplementary material for: Tuning Hierarchical Ferric Nanostructures-Decorated Diatomite for Supercapacitors
Source: Nanoscale Res Lett. 2018 Dec 18;13:407. doi: 10.1186/s11671-018-2822-9 (PMC6298910; doi:10.1186/s11671-018-2822-9)
Supplement: Supplementary file 1 — Experimental section. Figure S1. The SEM images of the samples in low magnification. Figure S2. (a) CV and (b) CC curves of the diatomite@MnO2 in 1 M Na2SO4; (c) CV and (d) CC curves of three kinds of ferric oxides-decorated diatomite. Figure S3. The EIS curve of Diatomite@FeOOH. Figure S4. The CV (a) curves at 100 mV s−1 and CC (b) curves at 1 A g−1 of diatomite, FeOOH and D@FeOOH. (DOC 22468 kb) [file 11671_2018_2822_MOESM1_ESM.doc]

Supplementary Information


SI-1. Experimental Section
SI-1.1 Materials synthesis
Purification of diatomite. The natural diatomite was refined via a simple oil bath method by the following procedures. First, the diatomite (5 g) was dissolved in deionized water (50 mL) with forcefully stirring, and the H2SO4 solution (50 g, 40 wt %) was dropwise added into the mixture at the same time. Second, the mixture was reacted in the round-bottom flask with constant magnetic stir (100 oC for 4 h). Afterwards, the mixture was stirred for another 1 h with 75 mL of deionized water added. Then, the acid-treated diatomite was washed with deionized water to get rid of the sulfuric acid during the filtration and dried at 60 oC overnight under air dry oven. Eventually, to remove residual organics and clean up the pores, the processed diatomite was heat-treated at 450 oC in a muffle stove.
Synthesis of ferric oxide-decorated diatomite composites. The MnO2-decorated diatomite composites were prepared by a facile hydrothermal method without any surfactant. The KMnO4 solution (30 mL, 0.05 M) serving as a homogeneous precursor was hybridized with the purified diatomite (30 mg). Afterwards, the mixture was removed into a Teflon-lined autoclave (50 mL) which was heat-treated at 160 oC for 24 h, then the resulting deep purple mixture was centrifuged, washed with distilled water and dried at 60 oC. After that, the as-prepared diatomite composites were calcined (200 oC for 2 h) in a muffle stove to stabilize the crystal form of the diatomite@MnO2. To obtain the ferric oxide-modified diatomite samples, a solution of FeSO47H2O (0.01 M, 30 mL) in mixed deionized water and ethylene glycol(volume ratio = 1 : 7) was transferred into Teflon-lined stainless steel autoclaves containing the pretreated diatomite@MnO2 (30 mg), which were maintained at 120 oC for 2 h. At this moment, diatomite@FeOOH composites were obtained. In the end, the different crystal forms (á-Fe2O3 and ã-Fe2O3) of ferric oxide-decorated diatomite were prepared by calcining at 350 oC for 2 h under O2 atmosphere and 500 oC for 2 h under N2 atmosphere, respectively.
SI-1.2 Electrochemical measurment
Before the electrochemical measurements, the working electrodes were prepared by mixing active material (diatomite@MnO2, diatomite@FeOOH, diatomite@á-Fe2O3 and diatomite@ã-Fe2O3), acetylene black and polyvinylidene fluoride (PVDF) at a weight ratio of 7:2:1 in N-methyl-2-pyrrolidone (NMP).The slurry was coated on a pieces of foamed nickel foam (1×1 cm2),which was heated to evaporate the dissolvent (120 oC for 12 h). The typical mass loading of the electrode material was 2 mg. The electrochemical performances and capacitance values of the composites electrodes were characterized with cyclic voltammetry (CV), galvanostatic charging/discharging methods (CC) and electrochemical impedance spectroscopy (EIS). 
The specific capacitance (Cm) is calculated by the following equation:
¡¡¡¡¡¡¡¡¡¡¡¡¡¡¡¡¡¡¡¡¡¡¡¡¡¡¡¡¡¡
where I is the discharging current, ¡÷t is the discharging time, ¡÷V is the potential window during¡¡discharging, and m is the weight of active materials.


SI-2 The SEM images of the samples in low magnification: diatomite@MnO2 (a), diatomite@FeOOH (b), diatomite@á-Fe2O3 (c), diatomite@ã-Fe2O3 (d); (e) the corresponding EDS mapping of diatomite@MnO2, (f) the corresponding EDS mapping of diatomite@FeOOH.


SI-3 (a) CV and (b) CC curves of the diatomite@MnO2 in 1 M Na2SO4; (c) CV curves of three kinds of ferric oxides-decorated diatomite measured at 100 mV s-1; (d) CC curves of three kinds of ferric oxides-decorated diatomite measured at 0.5 A g-1.


SI-4 The EIS curve of Diatomite@FeOOH (1M Na2SO4 solution).


SI-5 The CV (a) curves at 100 mV s-1 and CC (b) curves at 1 A g-1 of diatomite, FeOOH and D@FeOOH.
